# Radiology residency training in China: results from the first retrospective nationwide survey

**DOI:** 10.1186/s13244-021-00970-2

**Published:** 2021-02-17

**Authors:** Jingfeng Zhang, Xinxin Han, Zhenghan Yang, Zhenchang Wang, Jianjun Zheng, Zimo Yang, Jiming Zhu

**Affiliations:** 1 Department of Radiology, Hwa Mei Hospital, University of Chinese Academy of Sciences, Ningbo, China; 2 School of Medicine, Tsinghua University, Beijing, China; 3 Department of Radiology, Beijing Friendship Hospital, Capital Medical University, Beijing, China; 4 Vanke School of Public Health, Tsinghua University, Haidian District, Beijing, 100084 China

**Keywords:** Standardized residency training, Radiology residents, International medical education, ACGME six competencies, Heterogeneity

## Abstract

**Objectives:**

This was the first study to systematically landscape and examine China’s nationwide standardized residency training in radiology.

**Methods:**

In this retrospective cross-sectional study, we used data from the 2019 national survey of the first two cohorts of 3679 radiology residents who completed training in 2017 and 2018 across all 31 provinces in China. A total of 1163 (32%) residents participated in the survey. Multivariable logistic regression was used to examine the implementation frequency of 24 identified training tasks (categorized into six competencies) by region, demographics, and other residency information.

**Results:**

Among the 1163 respondents, 592 (51%) were trained in the more developed eastern region. Of the 24 identified training tasks, 15 were implemented significantly differently across regions, while the frequency of the most frequently conducted tasks (e.g., CT, MR, and radiograph interpretation and reporting) was consistent. The top 10 tasks all fell into the patient care and medical knowledge competency domains, while other competencies tended to be neglected. We found region and marital status were the most influential factors of training task implementation frequencies. Respondents trained in the northeast and the west were more likely to report, for instance, radiological examination recommendation (OR = 1.91, 95%CI = 1.27–2.88), as “very frequent.” Married respondents were more likely to report first-line night shift as “very frequent” (OR = 1.71, 95%CI = 1.29–2.26).

**Conclusions:**

Despite the fast-win achievements of developing a national radiology residency training program, there is a gap to train quality and homogeneous radiologists across regions. Future improvement should be more tailored to residents’ personal characteristics and emphasize some “soft” competencies (e.g., communication skills).

## Key points

- The multivariate regression analysis of the radiology residency programs in China indicates a wide training heterogeneity across country regions.
- China’s radiology residency training emphasizes patient care and medical knowledge, while the other four competencies such as professionalism and communication skills are paid less attention.
- Radiology residents’ some personal characteristics, such as marriage and gender, are significantly associated with the training task implementation.

## Introduction

Health care is essentially a labor-intensive sector, in which medical doctors play a leading role, and the residency system represents the dominant formative influence upon the “production” of these most valuable “assets” [1]. Since the Johns Hopkins initiated medical residency in 1889, the US residency system has well developed through over a century’s efforts [2]. In 2013, the US launched the Next Accreditation System, and this outcome-based reform highlighted the Six Core Competencies and relevant milestones developed by the Accreditation Council for Graduate Medical Education (ACGME) [3]. In China, medical residency can be traced back to the early twentieth century when the China Medical Board, an American foundation endowed by the Rockefeller family, established the Peking Union Medical College (PUMC) and the PUMC hospital, exactly following the Johns Hopkins model.

However, unlike the USA where the Johns Hopkins model was developed into a nationwide residency system, medical residency in China, for a long time, was only implemented rigorously in certain privileged hospitals. This has been changed since 2013 when China launched its nationwide standardized residency training (SRT) [4]. China’s SRT consists of 36 specialties, and radiology is one of them. Radiology was introduced to China together with the establishment of the PUMC when the American radiologist Paul C. Hodges, later widely recognized as the founder of radiology in China, became its first radiology director [5]. Though Dr. Hodges was sharply aware of the importance of radiology training and established a radiology school in China in 1920s, it was in 2017 that China finally had its first cohort of graduates from the nationwide SRT.

China’s SRT system is complicated, and a previous review has systematically analyzed it in comparison with its counterparts in the USA and UK [4]. When it comes to radiology residency, simply speaking, it takes three years to complete. More specifically, those who have graduated with a bachelor’s degree or above from a medical school (at least five years in China after high school) are required to complete the three-year residency in radiology. This SRT in radiology spans three years and contains three training stages. According to the contents and standards of China’s SRT [6], the first stage spans 15 months, and residents rotate in the radiology department (nine months), the ultrasound department (three months) and the department of nuclear medicine (three months). The second stage spans 18 months, and residents are based in the radiology department, going through the specific subspecialities. The third stage (the final three months) represents some flexible arrangements—residents can choose to carry out some research or continue rotating in their selected departments. During the three-year training, residents are required to master radiology-related clinical knowledge and skills and receive close supervisions and instructions from clinical faculty and senior radiologists. Upon the completion of the SRT, residents should have successfully passed the medical licensing examination and the residency certification examination (organized nationally). In the future, in order to be promoted as a staff radiologist and to undertake the full teaching responsibility, one must complete this SRT in radiology first.

Despite that China’s SRT in radiology has been implemented for seven years, evidence is still lacking on what specific tasks radiology residents actually undertake during the training. More importantly, one important goal of China’s SRT is to produce quality homogeneous doctors, but it is unclear whether radiology residency training has achieved consistency across the country. In 2019, exactly a century later when Dr. Hodges started to systematically promote radiology in China in 1919, the Chinese Association of Radiologists conducted a national cross-sectional survey to landscape and better characterize China’s SRT in radiology. The questionnaire was designed by following the conceptual framework of the ACGME Six Core Competencies. This article, accordingly, based on this retrospective national survey, aims to assess radiology residency training task implementation across regions and different groups of residents, as well as to shed insight on improving training quality and consistency across China.

## Methods

### Study design and participants

The Institutional Review Board at Research Center for Public Health, Tsinghua University, approved the study. This study used a retrospective national survey design. The national survey was conducted by the Chinese Association of Radiologists during August 16–31, 2019. The survey collected residency training-related information from residents who completed their residency training in 2017 or 2018 across all 31 provinces in China. These residents represent China’s first two cohorts of radiology residents who started their training in 2014 or 2015.

To ensure the quality and representativeness of the respondents, we defined the inclusion criterion of programs for this study as radiology residency programs that recruited 2 or more radiology residents in 2014 and 2015. A total of 308 (75%) of 408 radiology programs met this criterion. The directors of hospital radiology departments were firstly contacted by email or telephone, with a cover letter that clearly stated the purpose of the survey and the approach of survey distribution. The directors were asked to invite residents who completed residency training in 2017 or 2018 in their departments to participate in the survey. If a resident agreed to participate in the study, the survey was then distributed by the department director via the smartphone application (called “Wenjuanxing” in Chinese, a widely used online survey tool in China). Responding residents were asked to complete the questionnaire within one week after they were contacted. Participation of the survey was voluntary, and personal privacy was completely protected during the whole study process. All participants who are willing to complete the survey were asked for informed consents before taking the survey.

A total of 3,679 radiology residents completed residency training in 2017 or 2018 from the 308 surveyed residency programs, and 1,163 of them completed the questionnaire, yielding an overall response rate of 31.6%.

### Survey questionnaire

The questionnaire contains two parts. Part 1 of the questionnaire covered respondents’ demographic information, including their age, gender, degree, marital status and current working hospital tier, as well as their residency information, including years of completing residency, and residency training site name, province, bed size, and tier. Part 2 mainly included a 24-item self-administered questionnaire designed by the Chinese Association of Radiologists to assess the implementation frequency of routine residency training tasks (see Table 3 for a detailed list of the 24 items). The survey items were designed and selected based on the requirements and regulations of China’s radiology residency training. Survey respondents were asked to rate the implementation frequency of each training task on a five-point scale (numbered from 1 to 5), wherein “1” represents once a month or less, “2” represents 2 or 3 times a month, “3” represents 1 or 2 times a week, “4” represents 3 or 4 times a week, and “5” represents once a day or more.

### Dependent variable: residency training task implementation frequency

The primary outcome measure in this study was the probability of residents who reported a training task as “very frequent.” For this analysis, a task that was rated with a score of 4 or 5 was viewed as “very frequent.” We created 24 dummy variables for each identified training task, where 1 equal to respondents rated the task with a score of 4 or 5, otherwise 0.

### Independent variables

We included the following variables in the analysis: residents’ age (≥ 30 vs. < 30), gender (female vs. male), marital status (married vs. unmarried), degree (master/doctoral vs. bachelor) and current working hospital tier (tertiary vs. secondary) [7], as well as their residency completion year (2018 vs. 2017), residency training hospital size (≥ 3000 beds, 2000–2999 beds, vs. < 2000 beds) and residency training site region (central, west, northeast vs. east). China’s National Bureau of Statistics defined four regions based on the geographic location and economic status of each province. The eastern region contains 10 highly developed coastal provincial administrations, including Beijing, Tianjin, Hebei, Shanghai, Jiangsu, Zhejiang, Fujian, Shandong, Guangdong and Hainan. The central region includes 6 provinces, including Shanxi, Anhui, Jiangxi, Henan, Hubei and Hunan. The western region includes 12 less developed provincial administrations, including Inner Mongolia, Guangxi, Chongqing, Sichuan, Guizhou, Yunnan, Tibet, Shaanxi, Gansu, Qinghai, Ningxia and Xinjiang. The northeastern region consists of 3 provinces, including Liaoning, Jilin and Heilongjiang. We followed this classification to define the region of residency training sites.

### Geographic distribution of China’s radiology residency programs

**Fig. 1:**
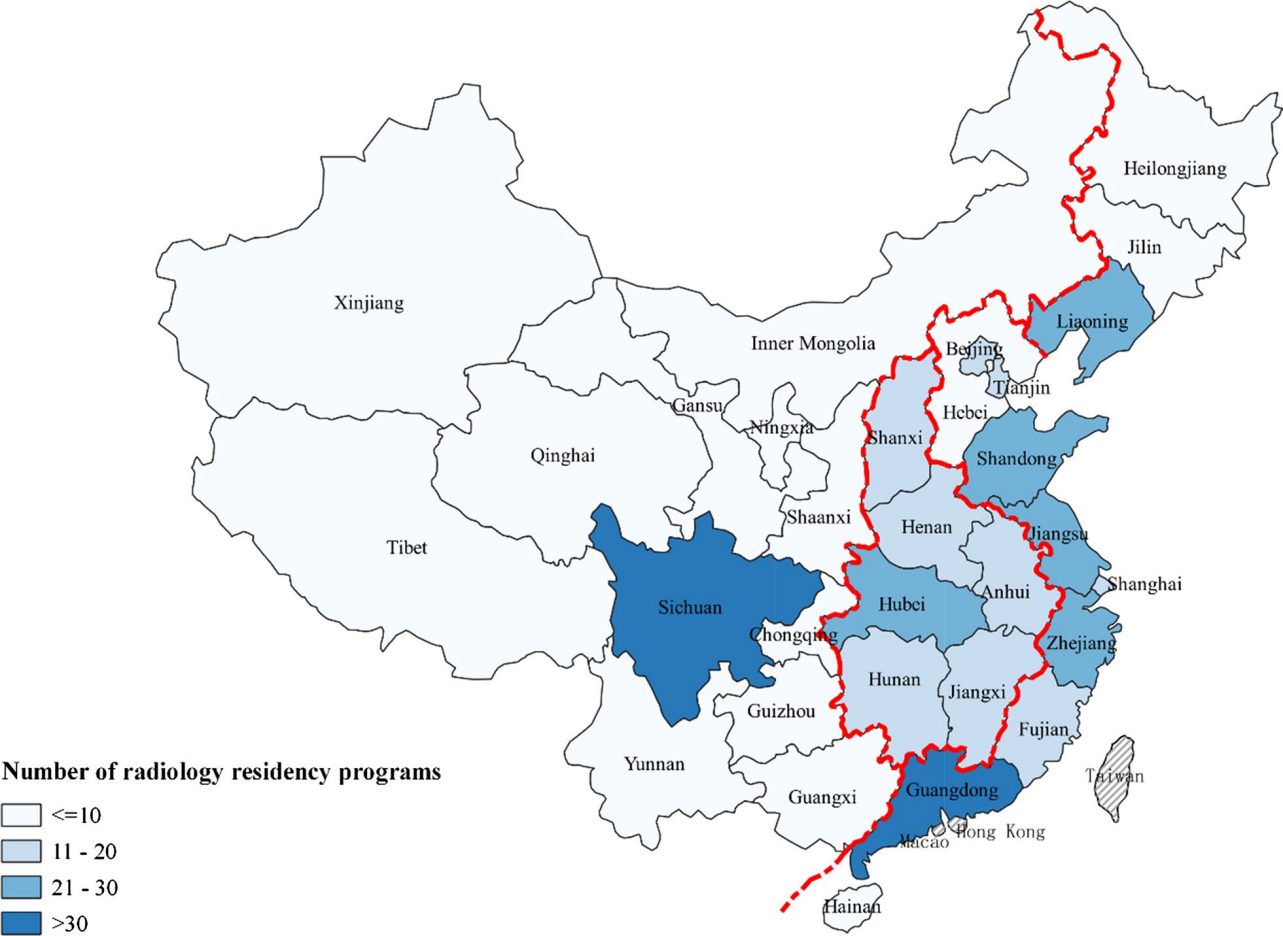
Geographic distribution of radiology residency programs in China. Note: The four regions were defined by China’s National Bureau of Statistics based on the geographic location and economic status. The eastern region contains 10 highly developed coastal provinces and municipalities, including Beijing, Tianjin, Hebei, Shanghai, Jiangsu, Zhejiang, Fujian, Shandong, Guangdong and Hainan. The central region includes 6 provinces, including Shanxi, Anhui, Jiangxi, Henan, Hubei and Hunan. The western region includes 12 less developed provinces and municipalities, including Inner Mongolia, Guangxi, Chongqing, Sichuan, Guizhou, Yunnan, Tibet, Shaanxi, Gansu, Qinghai, Ningxia and Xinjiang. The northeastern region consists of 3 provinces, including Liaoning, Jilin and Heilongjiang. Data on Macao, Hong Kong and Taiwan are not available

Figure 1 and Table 1 demonstrate the geographic distribution of radiology residency programs in China in 2014. Radiology residency programs generally concentrated on the eastern region (207 programs). The central region had 112 programs, the western region had 89 programs, and the northeastern region had 41 programs. More specifically, Guangdong (in the eastern region) and Sichuan (in the west region) had the highest number of radiology residency programs (36 and 31).

**Table 1:** Number of radiology residency programs and residents by region

Region	Number of residency programs	Number of surveyed programs	Number of residents in surveyed programs	Number of responding residents
Total	408	308	3679	1163
East	185	144	1879	592
Central	93	67	655	186
West	89	68	743	241
Northeast	41	29	402	144

The four regions were defined by China’s National Bureau of Statistics based on the geographic location and economic status. The eastern region contains 10 highly developed coastal provinces and municipalities, including Beijing, Tianjin, Hebei, Shanghai, Jiangsu, Zhejiang, Fujian, Shandong, Guangdong and Hainan. The central region includes 6 provinces, including Shanxi, Anhui, Jiangxi, Henan, Hubei and Hunan. The western region includes 12 less developed provinces and municipalities, including Inner Mongolia, Guangxi, Chongqing, Sichuan, Guizhou, Yunnan, Tibet, Shaanxi, Gansu, Qinghai, Ningxia and Xinjiang. The northeastern region consists of 3 provinces, including Liaoning, Jilin and Heilongjiang

### Statistical analysis

We began with a set of descriptive analyses. First, we mapped the geographic distribution of China’s first cohort of radiology residency programs (408 training sites) in 2014. Second, we adopted the ACGME Six Core Competencies as a conceptual framework to categorize the 24 identified tasks into 6 groups (see categorization in Table 3). The patient care and technical skills domain contains 9 tasks, the medical knowledge domain contains 5 tasks, the systems-based practice domain contains 4 tasks, and the rest three domains, i.e., interpersonal and communications skills, practice-based learning and improvement and professionalism, contain 2 tasks, respectively. We presented the percent of respondents who reported training tasks as “very frequent” for each task and rated the tasks based on percentages within each ACGME competency domain. We conducted Pearson's Chi-square tests to compare the percentages by region.

Because residents’ demographics (e.g., age, gender) and residency information may confound regional differences in reported frequencies, in the multivariable regression analysis, we estimated 24 logistic regression models of the probability of residents who reported a training task as “very frequent,” as a function of residents’ age, gender, marital status, degree, current working hospital tier, residency completion year, residency training hospital size and residency training site region. We also included in the models the time spent in completing the survey as a proxy for quality control of filling the survey. Standard errors were clustered by residency training hospital to account for autocorrelation among residents. We considered a *p* value of less than 0.05 to be statistically significant. All analyses were performed using Stata, version 16 (StataCorps, Inc.).

## Results

### Characteristics of study participants

The study participants consisted of 1163 radiology residents who completed their training in 2017 and 2018 from 308 radiology residency programs across all 31 provinces in China. Of these survey respondents, over half were below 30 years (55%), female (54%) and married (59%) (Table 2). About 48% hold a master’s degree and 4% hold a doctoral degree. The majority (71%) currently work in general tertiary A level hospitals, 8% work in specialist tertiary hospitals, 7% work in general tertiary B hospitals, 12% work in general secondary hospitals, and 2% work in specialist secondary hospitals. About 58% completed the residency training in 2018 vs 42% in 2017. About 97% residents were trained in general tertiary A hospitals, 3% were trained in specialist tertiary A hospitals, and only 1 resident was trained in a general secondary A hospital. Twenty percent were trained in super large hospitals (beds > 3000), and twenty percent were trained in relatively small hospitals (beds ≤ 1500). Over half (51%) were trained in hospitals in the eastern region, 16% were trained in the central region, 21% were trained in the western region, and the rest (12%) were trained in the northeastern region.

**Table 2:** Demographic and residency characteristics of responding radiology residents of 2014 and 2015 (*N* = 1,163)

	No	%
*Demographic information*		
Age		
< 30	641	55
≥ 30	522	45
Gender		
Male	531	46
Female	632	54
Marital status		
Unmarried	482	41
Married	681	59
Degree		
Bachelor	559	48
Master	555	48
Doctoral	49	4
Current working hospital tier		
General tertiary A	828	71
Specialist tertiary	88	8
General tertiary B	86	7
General secondary	134	12
Specialist secondary	27	2
*Residency information*		
Residency completion year		
2017	486	42
2018	677	58
Residency training site tier		
General tertiary A	1127	97
Specialist tertiary A	35	3
General secondary	1	0
Residency training site bed size		
≤ 1500 beds	231	20
1501–2000 beds	253	22
2001–2500 beds	277	24
2501–3000 beds	165	14
> 3000 beds	237	20
Region		
East	592	51
Central	186	16
West	241	21
Northeast	144	12

### Descriptive analysis

Table 3 shows the percent of respondents who reported a training task as “very frequent” (i.e., a score of 4 or 5) by region within the ACGME six competencies. In the patient care and technical skills domain, the first three highly rated tasks were CT interpretation and reporting (92% respondents rated it as “very frequent,” also the first highest rated task among all tasks), MR interpretation and reporting (84% rated it as “very frequent,” also the second highest rated task among all tasks) and radiograph interpretation and reporting (80% rated it as “very frequent”). There was no statistically significant difference in the percent of respondents who reported these tasks as “very frequent” across region. The last three cited tasks were barium X-rays (47%), first-line night shift (45%) and interventional procedures (only 27%, also the lowest rated tasks among all tasks). Reported frequencies of these three tasks varied differently across region.

**Table 3:** Descriptive analysis of the percent of respondents who reported a training task as “very frequent”

ACGME	Residency training task	Total (%)	Region				*p* value
Six core competencies			East (%)	Central (%)	West (%)	Northeast (%)	
Patient care and technical skills	CT interpretation & reporting	92	92	90	93	97	0.146
	MR interpretation & reporting	84	82	87	83	90	0.062
	Radiograph interpretation & reporting	80	80	77	84	79	0.326
	Reporting for emergency department	74	69	76	82	79	< *0.001*
	Radiological examination consultation	63	62	67	64	63	0.558
	Radiological examination recommendation	60	55	61	64	70	*0.003*
	Barium X-rays	47	44	55	49	48	*0.043*
	First-line night shift	45	39	47	54	54	< *0.001*
	Interventional procedures	27	24	25	31	33	*0.035*
Medical knowledge	Morning case conference	83	88	78	78	82	< *0.001*
	Challenging cases discussion	76	77	72	72	82	0.076
	Film-reading interactive lectures	69	69	69	64	79	*0.019*
	CT image Processing	66	63	73	66	73	*0.024*
	Didactic lectures	66	65	65	61	80	*0.001*
Systems-based practice	Pathology follow-up	62	58	63	66	65	0.131
	Report quality control	60	56	61	63	72	*0.007*
	Radiation safety	54	48	57	59	61	*0.003*
	Management of contrast media reactions	34	33	29	37	43	*0.029*
Interpersonal and communication skills	Clinical communication	63	59	65	67	72	*0.011*
	Supervising & teaching medical students/junior residents	55	53	56	55	67	*0.015*
Practice-based learning and improvement	Literature study	50	51	48	44	58	0.071
	Case report & paper writing	29	28	27	27	39	*0.043*
Professionalism	Group study & peer support	60	60	61	59	65	0.608
	Team building	44	43	45	43	51	0.302

A task that was rated with a score of 4 or 5 was viewed as “very frequent.” *p* values were from Pearson's Chi-square tests. The four regions were defined by China’s National Bureau of Statistics based on the geographic location and economic status. The eastern region contains 10 highly developed coastal provinces and municipalities, including Beijing, Tianjin, Hebei, Shanghai, Jiangsu, Zhejiang, Fujian, Shandong, Guangdong and Hainan. The central region includes 6 provinces, including Shanxi, Anhui, Jiangxi, Henan, Hubei and Hunan. The western region includes 12 less developed provinces and municipalities, including Inner Mongolia, Guangxi, Chongqing, Sichuan, Guizhou, Yunnan, Tibet, Shaanxi, Gansu, Qinghai, Ningxia and Xinjiang. The northeastern region consists of 3 provinces, including Liaoning, Jilin and Heilongjiang

*p* values smaller than 0.05 are indicated using italic

In the medical knowledge domain, the first three highly rated tasks were morning case conference (83%), challenging case discussion (76%) and film-reading interactive lectures (69%). Except for challenging case discussion, reported frequencies for other tasks under this domain varied across region. In the domain of systems-based practice, 34% respondents rated management of contrast media reactions as “very frequent,” and this was also the third lowest rated tasks among all tasks. Reported frequencies varied across region for report quality control, radiation safety and management of contrast media reactions.

For interpersonal and communication skills, nearly two-thirds (63%) respondents rated training on clinical communication as “very frequent” and over half (55%) rated activities involving supervising and teaching medical students or junior residents as “very frequent,” and their reported frequencies varied across region. For practice-based learning improvement, half (50%) of respondents rated literature study as “very frequent,” compared to only 29% respondents rated case report and paper writing as “very frequent” (also the second lowest rated task among all tasks). For professionalism, 60% respondents rated group study and peer support as “very frequent” and 44% rated team building as “very frequent.” Reporting frequencies for these two tasks did not vary across region.

### Regression analysis of the probability of respondents who reported a training task as very frequent

Table 4 presents the odds ratios (ORs) and 95% confidence intervals (CI) from the 24 multivariable logistic regression models. Consistent with the descriptive analysis, the probability of respondents reported a training task as “very frequent” varied largely by the region. Compared to respondents who were trained in eastern hospitals, respondents who were trained in the northeastern region were more likely to rate 12 tasks as “very frequent,” such as radiological examination recommendation (OR = 1.91, 95% CI = 1.27–2.88), first-line night shift (OR = 1.75, 95% CI = 1.30–2.36) and supervising and teaching medical students or junior residents (OR = 1.97, 95%CI = 1.45–2.70). Respondents who were trained in the west were more likely to rate 6 tasks, such as reporting for emergency department (OR = 1.90, 95% CI = 1.26–2.86) and first-line night shift (OR = 1.97, 95%CI = 1.37–2.85), as “very frequent.” Respondents who were trained in the central region were more likely to rate 2 tasks, i.e., barium X-rays (OR = 1.56, 95% CI = 1.09–2.23) and CT image processing (OR = 1.60, 95% CI = 1.00–2.54), as “very frequent.” Interestingly, compared to the east, respondents who were trained in the central and the west were less likely to rate morning case conference as “very frequent” (OR for east = 0.47, 95% CI = 0.27–0.81; OR for west = 0.49, 95% CI = 0.28–0.86), while no statistically significant difference was found between respondents trained in the east and northwest (OR = 0.58, 95% CI = 0.33–1.01). We also found that respondents who were married were more likely to rate 11 tasks, such as first-line night shift (OR = 1.71, 95% CI = 1.29–2.26), management of contrast media reactions (OR = 1.57, 95% CI = 1.17–2.10) and reporting for emergency department (OR = 1.51, 95% CI = 1.12–2.03), as “very frequent” than unmarried respondents.

**Table 4:** Multivariable logistic regression analysis of the probability of respondents reporting a training task as “very frequent”

Demographic and residency characteristics	Patient care and technical skills								
	CT interpretation & reporting	MR interpretation & reporting	Radiograph interpretation & reporting	Reporting for emergency department	Radiological examination consultation	Radiological examination recommendation	Barium X-rays	First-line night shift	Interventional procedures
Age ≥ 30 (vs. age < 30)	0.96	1.21	1.21	1.28	1.34	1.10	1.11	1.16	1.20
	(0.59–1.56)	(0.86–1.69)	(0.87–1.69)	(0.95–1.73)	(1.04–1.73)	(0.85–1.42)	(0.87–1.41)	(0.90–1.51)	(0.90–1.61)
Female (vs. male)	1.17	0.99	0.93	0.96	1.16	0.76	0.74	0.74	0.54
	(0.76–1.82)	(0.70–1.38)	(0.68–1.28)	(0.73–1.25)	(0.89–1.52)	(0.59–0.98)	(0.58–0.93)	(0.57–0.95)	(0.41–0.70)
Married (vs. unmarried)	1.53	1.50	1.26	1.51	1.32	1.43	1.33	1.71	1.07
	(0.97–2.39)	(1.03–2.18)	(0.89–1.78)	(1.12–2.03)	(1.01–1.73)	(1.09–1.86)	(1.01–1.75)	(1.29–2.26)	(0.79–1.44)
Master/doctoral degree (vs. bachelor)	1.12	1.31	0.77	0.79	0.91	1.10	0.88	1.27	1.21
	(0.66–1.90)	(0.89–1.92)	(0.53–1.12)	(0.57–1.09)	(0.67–1.24)	(0.83–1.45)	(0.69–1.12)	(0.96–1.67)	(0.88–1.67)
Current working hospital tier: tertiary (vs. non-tertiary)	0.85	1.09	1.02	1.61	0.90	1.23	1.23	1.41	1.30
	(0.40–1.78)	(0.68–1.75)	(0.64–1.64)	(1.09–2.36)	(0.62–1.32)	(0.86–1.76)	(0.83–1.81)	(0.92–2.16)	(0.80–2.13)
Residency completion year: 2018 (vs. 2017)	1.42	1.22	1.21	1.10	1.13	1.14	1.26	1.28	1.00
	(0.90–2.24)	(0.84–1.78)	(0.88–1.66)	(0.81–1.51)	(0.89–1.43)	(0.89–1.48)	(1.00–1.58)	(0.98–1.66)	(0.76–1.32)
Residency training site bed size: 2000–2999 beds (vs. < 2000 beds)	1.08	0.91	0.84	0.95	0.85	0.89	0.88	0.95	1.07
	(0.64–1.83)	(0.57–1.45)	(0.56–1.26)	(0.65–1.38)	(0.64–1.15)	(0.66–1.20)	(0.64–1.20)	(0.69–1.31)	(0.75–1.53)
Residency training site bed size: ≥ 3000 beds (vs. < 2000 beds)	1.55	1.86	0.96	1.10	1.05	0.85	0.70	0.90	1.05
	(0.83–2.91)	(1.08–3.20)	(0.61–1.52)	(0.72–1.66)	(0.74–1.51)	(0.60–1.22)	(0.49–0.98)	(0.65–1.26)	(0.71–1.56)
*Residency training site region*									
Central (vs. East)	0.79	1.33	0.77	1.25	1.20	1.24	1.56	1.39	1.05
	(0.42–1.49)	(0.81–2.20)	(0.48–1.24)	(0.79–1.99)	(0.86–1.68)	(0.86–1.79)	(1.09–2.23)	(0.95–2.02)	(0.67–1.63)
West (vs. East)	1.23	1.10	1.21	1.90	1.08	1.47	1.15	1.97	1.53
	(0.63–2.40)	(0.65–1.87)	(0.77–1.89)	(1.26–2.86)	(0.72–1.63)	(1.02–2.11)	(0.82–1.61)	(1.37–2.85)	(1.06–2.22)
Northeast (vs. East)	2.38	1.64	0.96	1.58	1.01	1.91	1.25	1.75	1.54
	(1.00–5.66)	(0.86–3.13)	(0.58–1.59)	(0.78–3.23)	(0.70–1.47)	(1.27–2.88)	(0.84–1.87)	(1.30–2.36)	(0.94–2.49)

Demographic and residency characteristics	Medical knowledge					Systems-based practice			
	Morning case conference	Challenging cases discussion	Film-reading interactive lectures	CT image processing	Didactic lectures	Pathology follow-up	Report quality control	Radiation safety	Management of contrast media reactions
Age ≥ 30 (vs. age < 30)	0.95	0.88	0.94	1.25	0.75	1.10	1.18	1.39	1.16
	(0.65–1.39)	(0.66–1.18)	(0.72–1.24)	(0.95–1.65)	(0.58–0.97)	(0.85–1.41)	(0.91–1.53)	(1.09–1.77)	(0.88–1.53)
Female (vs. male)	1.00	0.93	0.83	0.99	0.70	0.89	0.92	0.95	0.83
	(0.70–1.43)	(0.70–1.23)	(0.64–1.08)	(0.76–1.28)	(0.55–0.90)	(0.70–1.13)	(0.73–1.16)	(0.74–1.21)	(0.65–1.06)
Married (vs. unmarried)	1.33	1.29	1.43	1.26	1.39	1.17	1.11	1.28	1.57
	(0.94–1.88)	(0.95–1.75)	(1.09–1.89)	(0.97–1.64)	(1.05–1.84)	(0.92–1.49)	(0.86–1.43)	(0.96–1.71)	(1.17–2.10)
Master/doctoral degree (vs. bachelor)	1.16	1.60	1.06	1.19	1.12	1.28	1.01	0.87	1.22
	(0.79–1.70)	(1.17–2.17)	(0.79–1.41)	(0.88–1.60)	(0.84–1.50)	(0.97–1.69)	(0.75–1.35)	(0.65–1.15)	(0.92–1.60)
Current working hospital tier: tertiary (vs. non-tertiary)	0.87	0.84	1.21	0.95	1.05	1.35	1.11	1.10	1.03
	(0.49–1.54)	(0.55–1.27)	(0.84–1.74)	(0.63–1.42)	(0.71–1.56)	(0.95–1.93)	(0.77–1.61)	(0.74–1.62)	(0.68–1.56)
Residency completion year: 2018 (vs. 2017)	0.84	1.30	1.13	0.99	1.15	1.13	1.10	0.99	1.33
	(0.62–1.14)	(0.98–1.73)	(0.87–1.47)	(0.76–1.29)	(0.87–1.53)	(0.88–1.44)	(0.85–1.43)	(0.76–1.29)	(1.04–1.70)
Residency training site bed size: 2000–2999 beds (vs. < 2000 beds)	1.21	0.95	1.06	0.96	1.18	1.21	0.93	0.86	1.15
	(0.78–1.89)	(0.66–1.36)	(0.79–1.42)	(0.68–1.35)	(0.86–1.61)	(0.88–1.67)	(0.68–1.28)	(0.63–1.17)	(0.84–1.57)
Residency training site bed size: ≥ 3000 beds (vs. < 2000 beds)	1.62	0.78	1.30	0.83	1.37	1.06	0.93	0.85	0.77
	(0.91–2.89)	(0.50–1.20)	(0.86–1.95)	(0.57–1.21)	(0.90–2.08)	(0.73–1.54)	(0.64–1.36)	(0.60–1.22)	(0.55–1.08)
*Residency training site region*									
Central (vs. East)	0.47	0.82	0.91	1.60	0.94	1.27	1.19	1.35	0.85
	(0.27–0.81)	(0.53–1.28)	(0.57–1.45)	(1.00–2.54)	(0.60–1.47)	(0.87–1.85)	(0.81–1.77)	(0.97–1.88)	(0.58–1.25)
West (vs. East)	0.49	0.82	0.76	1.16	0.81	1.46	1.29	1.51	1.23
	(0.28–0.86)	(0.54–1.24)	(0.54–1.07)	(0.78–1.73)	(0.57–1.15)	(0.99–2.16)	(0.87–1.92)	(1.03–2.23)	(0.88–1.71)
Northeast (vs. East)	0.58	1.40	1.59	1.58	2.14	1.27	1.90	1.65	1.63
	(0.33–1.01)	(0.85–2.32)	(1.13–2.25)	(1.08–2.31)	(1.27–3.62)	(0.85–1.91)	(1.35–2.68)	(1.13–2.42)	(1.15–2.31)

Demographic and residency characteristics	Interpersonal and communication skills		Practice-based learning and improvement		Professionalism	
	Clinical Communication	Supervising & teaching medical students/junior residents	Literature study	Case report & paper writing	Group study & peer support	Team building
Age ≥ 30 (vs. age < 30)	1.26	1.13	1.10	0.98	1.12	1.21
	(0.97–1.64)	(0.88–1.45)	(0.85–1.42)	(0.75–1.30)	(0.87–1.44)	(0.92–1.59)
Female (vs. male)	0.92	0.95	0.99	0.77	0.75	0.84
	(0.72–1.18)	(0.74–1.22)	(0.78–1.26)	(0.59–1.01)	(0.59–0.95)	(0.66–1.07)
Married (vs. unmarried)	1.42	1.34	1.19	1.16	1.23	1.27
	(1.09–1.86)	(1.03–1.75)	(0.92–1.52)	(0.87–1.55)	(0.95–1.60)	(0.98–1.64)
Master/doctoral degree (vs. bachelor)	1.24	1.05	1.48	1.54	1.27	1.47
	(0.93–1.64)	(0.80–1.37)	(1.13–1.95)	(1.11–2.14)	(0.97–1.66)	(1.13–1.91)
Current working hospital tier: tertiary (vs. non-tertiary)	1.09	1.07	1.06	1.37	1.17	1.45
	(0.75–1.57)	(0.75–1.52)	(0.76–1.47)	(0.88–2.13)	(0.82–1.68)	(1.00–2.11)
Residency completion year: 2018 (vs. 2017)	1.06	1.10	1.21	1.07	1.16	1.15
	(0.82–1.38)	(0.87–1.38)	(0.95–1.55)	(0.81–1.41)	(0.89–1.51)	(0.90–1.47)
Residency training site bed size: 2000–2999 beds (vs. < 2000 beds)	0.81	0.89	1.05	1.15	1.16	1.03
	(0.60–1.09)	(0.66–1.22)	(0.80–1.38)	(0.84–1.57)	(0.88–1.53)	(0.77–1.39)
Residency training site bed size: ≥ 3000 beds (vs. < 2000 beds)	0.73	0.68	0.99	1.00	1.25	0.95
	(0.50–1.07)	(0.47–0.97)	(0.71–1.39)	(0.70–1.43)	(0.92–1.70)	(0.67–1.36)
*Residency training site region*						
Central (vs. East)	1.26	1.17	0.93	1.02	1.03	1.10
	(0.90–1.77)	(0.82–1.68)	(0.65–1.31)	(0.70–1.48)	(0.73–1.46)	(0.76–1.59)
West (vs. East)	1.48	1.08	0.80	1.13	1.01	1.05
	(1.02–2.13)	(0.75–1.55)	(0.58–1.11)	(0.76–1.66)	(0.73–1.41)	(0.76–1.45)
Northeast (vs. East)	1.75	1.97	1.24	1.60	1.19	1.30
	(1.11–2.75)	(1.45–2.70)	(0.89–1.71)	(1.13–2.27)	(0.85–1.65)	(0.92–1.84)

Odds ratios and 95% confidence intervals were presented

In contrast, the probability of residents reported a task as “very frequent” varied less by other demographic and residency characteristics. Some interesting findings are that females compared to males were less likely to rate a number of tasks, such as interventional procedures (OR = 0.54, 95% CI = 0.41–0.70), as “very frequent.” Compared to those who hold a bachelor’s degree, respondents who hold a master’s or doctoral degree were more likely to rate challenging cases discussion (OR = 1.60, 95% CI = 1.17–2.17), literature study (OR = 1.48, 95% CI = 1.13–1.95) and case report and paper writing (OR = 1.54, 95% CI = 1.11–2.14) as “very frequent.” Respondents who currently work in tertiary hospitals were 1.61 (95% CI = 1.09–2.36) times more likely to rate reporting for emergency department as “very frequent” than respondents who currently work in secondary hospitals.

## Discussion

China has used only several years to establish a nationwide functioning residency training system. For the first time, all radiology residency programs across the country have been generally standardized. The SRT programs currently emphasize patient care and medical knowledge, while the other four competency domains such as professionalism should be improved. The design and improvement of the SRT should consider residents’ personal characteristics such as marriage and gender, as they can be significantly associated with some training tasks. Also, there is a wide training heterogeneity across the country, which arguably poses the biggest threat to China’s newly established SRT system.

Our study reveals that the daily routine of China’s radiology residents is mostly CT, MR, and radiograph interpretation & reporting, morning case conference, and challenging cases discussion. Among these five most frequently conducted activities, four have no significant difference across regions. Past studies indicated that the key components of radiology expertise included medical knowledge, visual skills such as recognition of patterns, and interpretation schemes [8]. Our findings suggest that radiology residency training in China is generally on the right track of developing radiologists’ expertise, and to some extent, the routine training is consistent across China. In addition, our findings indicate that radiology residency training in China pays special attention to two core competencies—patient care and technical skills, and medical knowledge. Among the 24 training tasks examined in this study, the top 10 most frequently conducted tasks all fall into these two domains. Patient care and medical knowledge are obviously requisite, while the other four competency domains which more relate to “soft” skills should be strengthened in China’s radiology residency training. In particular, interpersonal and communication skills are vital for patient outcomes [9] and doctor–patient relationships [10]. However, past studies revealed that interpersonal and communication skills are often neglected in the diagnostic process among China’s doctors [11], although communication skills can be effectively enhanced through designated training [12, 13]. Also, the cultivation of professionalism in China is consistently low across region, and this supports the lack of a widely shared tradition of medical professionalism in China as a foundation for modernized health systems [14].

In China, the discipline of medical imaging includes radiology, ultrasound and nuclear medicine, yet the residency training in radiology, ultrasound, and nuclear medicine is in fact three separate training programs. In other words, China’s SRT consists of 36 specialties, and radiology, ultrasound and nuclear medicine are three parallel specialties. Although radiology residents are required to rotate in ultrasound and nuclear medicine departments, the requirements for the ultrasound and nuclear medicine training in radiology residency are not that strict. From the medical education perspective, better integration and coordination of radiology, ultrasound and nuclear medicine are important. In addition, for China’s SRT in radiology, the training in interventional radiology tends to be neglected. According to contents and standards of China’s radiology residency training, there is a minimum requirement that residents should complete 30 cases of interventional procedure observations during the training, but there are no specific requirements regarding conducting interventional procedures. Also, because rotation to interventional diagnosis and treatment part depends on personal preferences, residents can selectively rotate or not. This has been reflected from our findings that interventional procedures are the lowest rated task among all training tasks, which signals that future training in interventional radiology needs to be improved.

Some personal characteristics of the residents are also significantly associated with the reported activities of the radiology residency training. Respondents’ marital status is the most influential factor. About two-thirds of survey respondents were married, and they carried out all the training activities more frequently than those unmarried—all odds ratios from the 24 models were above 1 and 12 were significant. Though our analysis is not sufficient to figure out the mechanism of this “influential” association, it appears to be consistent with international experiences. A prior study has found that in the USA, married radiology residents are more likely to feel personal accomplishment than unmarried residents [15], which is likely to be related to work tasks they undertook during residency. In addition, female residents appear to implement training tasks generally less frequently than male residents. The least conducted task is interventional procedures, and the likelihood of female residents reporting interventional procedures as “very frequent” is further reduced significantly to a half, compared to male residents. This is consistent with the findings in the USA—female represented less than 10% of all interventional radiologists nationally [16], and among radiology residents, female accounts for only 12% [17].

One purpose of China’s SRT is to train homogeneous doctors. However, our study shows that heterogeneity still widely exists in China. Firstly, radiology residency programs are not evenly distributed across regions, with the more developed eastern region having the most residency programs. This echoes the maldistribution of radiologists around the country [18]. In the USA, radiologists and radiology resident workforce also present a maldistribution across states and rural–urban areas, suggesting a need of geographic redistribution rather than simply increasing the overall number [19]. Residents are also an important workforce, as they would immediately increase clinical capacity of their training sites and ultimately expand the workforce [20]. Accordingly, the uneven distribution of training sites and resources may further worsen the maldistribution of radiologists in China. Secondly, except for the several most frequently conducted tasks, implementation frequencies of most training tasks varied across regions. For the 24 examined tasks, 15 are implemented significantly differently across the country. These variations create challenges to homogenize the residency training of radiologists in China, making the goal of China’s SRT harder to achieve.

This is the first national study to systematically examine China’s radiology residency training, and we acknowledge several limitations. First, this is a cross-sectional observational study, and therefore, no causality can be inferred. Second, the frequencies of residency training tasks are self-reported by radiology residents who have already graduated, and accordingly, like all retrospective studies, our estimates could be overestimated or underestimated due to recall bias and self-reported bias. Third, we have adopted the conceptual framework of the ACGME six competencies, but do not go into the detailed milestones. To address these limitations, a new nationwide study, using the latest milestones and targeting current residents in training, will be conducted. All the efforts will contribute to high-quality and homogeneous radiologists with fair distribution, and China’s experiences in comparison with the relatively mature US model may benefit other areas around the world, especially middle- and low-income countries, that are also making efforts to establish or improve residency training.

In conclusion, China has established a functioning nationwide standardized residency training system covering 408 radiology programs. Despite the fast-win achievements, there is a gap to train quality and homogeneous radiologists across regions. Future improvement should be more tailored to residents’ personal characteristics and emphasize some “soft” competencies such as communication skills.

## Data Availability

The data that support the findings of this study are available from the Chinese Association of Radiologists, but restrictions apply to the availability of these data, which were used under license for the current study, and so are not publicly available. Data are however available from the authors upon reasonable request and with permission of the Chinese Association of Radiologists.

## Abbreviations

ACGME: Accreditation Council for Graduate Medical Education
PUMC: Peking Union Medical College
SRT: Standardized residency training
MR: Magnetic resonance
CT: Computed tomography
